# Ethnic Disparities in Emergency Severity Index Scores among U.S. Veteran’s Affairs Emergency Department Patients

**DOI:** 10.1371/journal.pone.0126792

**Published:** 2015-05-29

**Authors:** Jacob M. Vigil, Joe Alcock, Patrick Coulombe, Laurie McPherson, Mark Parshall, Allison Murata, Heather Brislen

**Affiliations:** 1 University of New Mexico, Albuquerque, New Mexico, United States of America; 2 New Mexico Veterans Affairs Health Center, Albuquerque, New Mexico, United States of America; Swinburne University of Technology, AUSTRALIA

## Abstract

**Background:**

The goal of these analyses was to determine whether there were systematic differences in Emergency Severity Index (ESI) scores, which are intended to determine priority of treatment and anticipate resource needs, across categories of race and ethnicity, after accounting for patient-presenting vital signs and examiner characteristics, and whether these differences varied among male and female Veterans Affairs (VA) ED patients.

**Methods and Findings:**

We used a large national database of electronic medical records of ED patients from twenty-two U.S. Department of Veterans Affairs ED stations to determine whether ESI assignments differ systematically by race or ethnicity. Multi-level, random effects linear modeling was used to control for demographic characteristics and patient’s vital signs (heart rate, respiratory rate, and pain level), as well as age, gender, and experience of triage nurses. The dataset included 129,991 VA patients presenting for emergency care between 2008 and 2012 (91% males; 61% non-Hispanic White, 28% Black, 7% Hispanic, 2% Asian, <1% American Indian/Alaska Native, 1% mixed ethnicity) and 774 nurses for a total of 359,642 patient/examiner encounters. Approximately 13% of the variance in ESI scores was due to patient characteristics and 21% was due to the nurse characteristics. After controlling for characteristics of nurses and patients, Black patients were assigned less urgent ESI scores than White patients, and this effect was more prominent for Black males compared with Black females. A similar interaction was found for Hispanic males. It remains unclear how these results may generalize to EDs and patient populations outside of the U.S. VA Health Care system.

**Conclusions:**

The findings suggest the possibility that subgroups of VA patients receive different ESI ratings in triage, which may have cascading, downstream consequences for patient treatment quality, satisfaction with care, and trust in the health equity of emergency care.

## Introduction

Emergency Severity Index (ESI) scores and similar triage categorization systems have been widely used to determine the urgency and priority of patient treatment in the emergency department (ED). These systems are intended to rely on objective vital signs and presenting symptoms such as pain to prioritize care and anticipate resource needs for ED patients at the time of presentation, according to the severity of patients’ conditions. ESI scores have been shown to reliably predict hospital resources needed by ED patients, as well as the probability and location of hospital admission [1,2]. Numerous health care systems including the Veterans Health Administration (VHA) have mandated policies for ED Registered Nurses (RNs) to use ESI scores to triage patients who present for ED care.

Several studies have shown that African Americans and Hispanic ED patients, in particular, tend to wait significantly longer, are less likely to receive opioids, and are less likely to be prescribed analgesics on discharge than White patients presenting similar conditions (e.g., bone fractures, nontraumatic dental conditions [3–8]). There are mixed findings on whether patients with different ethnicities receive disparate ESI scores in U.S. EDs [9,10]; however, previous investigations were limited by statistical or other methodological issues, notably small sample size. In most cases, the visit has been the unit of analysis. Therefore, previous studies did not capture individual-level characteristics of patients and examiners and did not adequately account for multiple visits by the same patient or multiple triage score assignments by the same nurse. Until the present study, it has been unknown whether ESI scores differ according to patient race and ethnicity, after controlling for demographic characteristics and patient-presenting vital signs (heart rate [HR], respiratory rate [RR], and pain level), and basic demographic characteristics of triage nurses.

## Methods

### Study Population

We used a national database of U.S., Veteran’s Affairs (VA) ED patients to calculate whether race and ethnicity independently influenced the ESI scores assigned by triage nurses during visits between January 2008 and December 2012. The study was approved by the University of New Mexico Institutional Review Board and the New Mexico VA Health Care System Research and Development Committee and patient records/information was anonymized and de-identified prior to analysis. Data tables were sourced from the VA Corporate Data Warehouse, the operational database that houses the electronic medical records of US VA patients. Information from the patients and nurses who assigned the ESI scores to the patients were managed and combined via VA Informatics and Computing Infrastructure (VINCI); see S1 Supplemental Materials for a full description of data filtering, management, and manipulation procedures. Patients were categorized into one of six self-indicated ethnic/racial categories: non-Hispanic White, Hispanic, Black, Asian, American Indian/Alaskan Native, or mixed ethnicity.

### Study Design

Only VA hospitals (*n* = 22) using identifiable ESI scores were included in the analyses (see S1 Supplemental Materials). All the patients were U.S. veterans and were included in the study if their medical records indicated that they: 1) visited a VA ED facility at least once during a 5-year period between the dates of January 1^st^, 2008 and December 31^st^, 2012; 2) had been treated for an inflammatory or a musculoskeletal condition (broad pain-related categories); 3) were processed by a VA staff member with a nursing license (e.g., RN, LPN / LVN, NP); and 4) had complete vital signs and demographic data and information about their triage nurse. Only patients with an ESI score between 2 (representing time sensitive and high risk patient conditions) and 5 (low acuity and low anticipated resource needs) were considered in the analyses. Visits in which patients were assigned an ESI score of 1 were excluded, because this score is usually restricted to patients who require immediate resuscitation (e.g., who are unconscious or who have an immediately life-threatening condition), and in such cases there is little to no discretion to assign any but the most emergent ESI rating.

The primary dependent variable was patients’ ESI scores for individual visits. Additional patient characteristics included their gender, age, and the sum of distinct ICD9 (‘red flag’) codes indicating a current alcohol or substance-related diagnosis (behavior problems), and the vital signs: heart rate, respiratory rate, and patient-reported pain score (on a 0–10 scale). Nursing characteristics consisted of gender, age, and number of years of experience working for the VA. Visits with any missing patient or nurse information were removed from the dataset prior to analysis. Visits with outlying vital measurements for patients (i.e., heart and respiratory rates) were also excluded from analyses (see S1 Supplemental Materials).

Patient ethnicity was dummy-coded (using White as the reference group), as were patient and nurse gender (with women as the reference group in both cases). Continuous predictors were centered at the mean across visits (nurse age = 47.5, nurse experience = 14.0, heart rate = 81.4, respiratory rate = 18.3, pain score = 4.5, patient age = 59.5), except for problem behaviors (sum of alcohol/substance disorders), which was left centered at the modal value of 0.

The goal of these analyses was to determine whether there were systematic differences in ESI scores across categories of race and ethnicity, after accounting for other patient and examiner characteristics, and whether these differences varied among male and female ED patients. We used linear cross-classified, random-effects (multilevel) models [11] in order to account for the fact that some patients had multiple ED visits and most nurses provided multiple ESI ratings to different (and sometimes the same) patients within the 5-year period. Each ESI score was assigned to a particular patient by a particular nurse, but both nurses and patients potentially contributed multiple ESI scores. Random-effects models are preferred in this type of situation, because they are robust to violations of assumptions that observations and errors are independent.

In our models, each ESI score is nested under a specific nurse and a specific patient. This allows for the mean ESI score to vary across nurses (who may vary in their approach to ESI assessment, regardless of the particular patient they are evaluating) and patients (who may vary in their presenting complaints and communication styles). We estimated a model for all patient encounters that met our inclusionary criteria. In S1 Supplemental Materials we ran separate models for patients with different presenting conditions and patients in each U.S census region. In all random-effects models, the ESI score for a given visit is the dependent variable, and we included the dummy codes for patient ethnicity and patient gender as predictors, along with their interaction terms. We included as covariates patient characteristics that are expected to influence ESI scores (e.g., heart rate, respiratory rate, pain score, and substance abuse disorders) and nurse characteristics that should not (gender, age). We also included patient age and nurses’ years of experience because, under some circumstances, they might legitimately be relevant to triage decision-making. We conducted our analyses in R v3.1.0 [12] using restricted maximum-likelihood estimation in the package lme4 v1.1–6 [13]. To examine significant interactions, we used an online tool designed to decompose two-way interactions in random-effects models [14].

## Results

In total, the sample consisted of 129,991 patients with complete (patient and examiner) data for inclusion in the study. Since all patients were veterans, most patients (91% of the sample) were males. Patient age ranged from 18-103yrs (*M* = 58.3yrs, *SD* = 15.7). Each patient was seen between 1 to 157 times on separate visits by one of 774 nurses (78% female) across a total number of 359,642 visits/triage assignments (78% of patients had 1 to 3 total visits during the 5 year-period analyzed). The staff who assigned the ESI scores to the patients ranged in age from 21 to 75 years (*M* = 45.3, *SD* = 11.1) and assigned multiple ESI scores over the 5-year period analyzed (median = 16, 25% and 75% = [3.00, 287.25]). At the time of each assessment, the staff had between 0 and 43 years of experience (*M* = 11.6, *SD* = 9.0) since their initial employment with the VA Health Care System.

Table 1 shows the distribution of ESI scores in each racial or ethnic group, without accounting for nested, repeated measures. Relative to non-Hispanic White patients, each group except for American Indian/Alaska Native had an increased likelihood of being assigned a non-urgent ESI score (4 or 5).

**Table 1:** Number (percentage) of visits in each ESI score category (2 through 5) by race / ethnicity with odds ratio (OR, 95% CI) of receiving a non-urgent ESI score (4 or 5).

	ESI score					
	2	3	4	5	Total visits	OR* [95% CI]
AIAN	166 (10%)	691 (43%)	607 (38%)	127 (8%)	1,591	1.04 [0.94, 1.14]
Asian	524 (10%)	1,913 (35%)	2,506 (46%)	455 (8%)	5,398	1.47 [1.39, 1.55]
Black	8,483 (7%)	47,193 (39%)	51,398 (43%)	12,803 (11%)	119,877	1.39 [1.37, 1.41]
Hispanic	2,065 (9%)	9,967 (42%)	9,615 (41%)	1,807 (8%)	23,454	1.15 [1.12, 1.18]
Mixed	396 (8%)	2,234 (43%)	2,088 (41%)	427 (8%)	5,145	1.16 [1.09, 1.22]
White	18,192 (9%)	93,541 (46%)	77,248 (38%)	15,196 (7%)	204,177	1.00
Total	29,826 (8%)	155,539 (43%)	143,462 (40%)	30,815 (9%)	359,642	

ESI Emergency Severity Index; AIAN American Indian / Alaska Native.

*Reference category is non-Hispanic White. Each odds ratio indicates how much more likely each minority group is to be assigned a non-urgent ESI score (4 or 5) relative to White patients. For example, Asian patients are about one and a half (1.47) times more likely to be assigned a non-urgent ESI score than White patients.

We computed the proportions of variance in ESI scores attributable to differences across patients and nurses (for additional detail, see S1 Supplemental Materials). We examined whether racial or ethnic differences in ESI scores remained after accounting for differences in age, heart and respiratory rate, pain score, behavioral problems, nurse gender, nurse age, and nurse experience. We also examined whether racial or ethnic differences, differed depending on patient gender.

**Fig 1:**
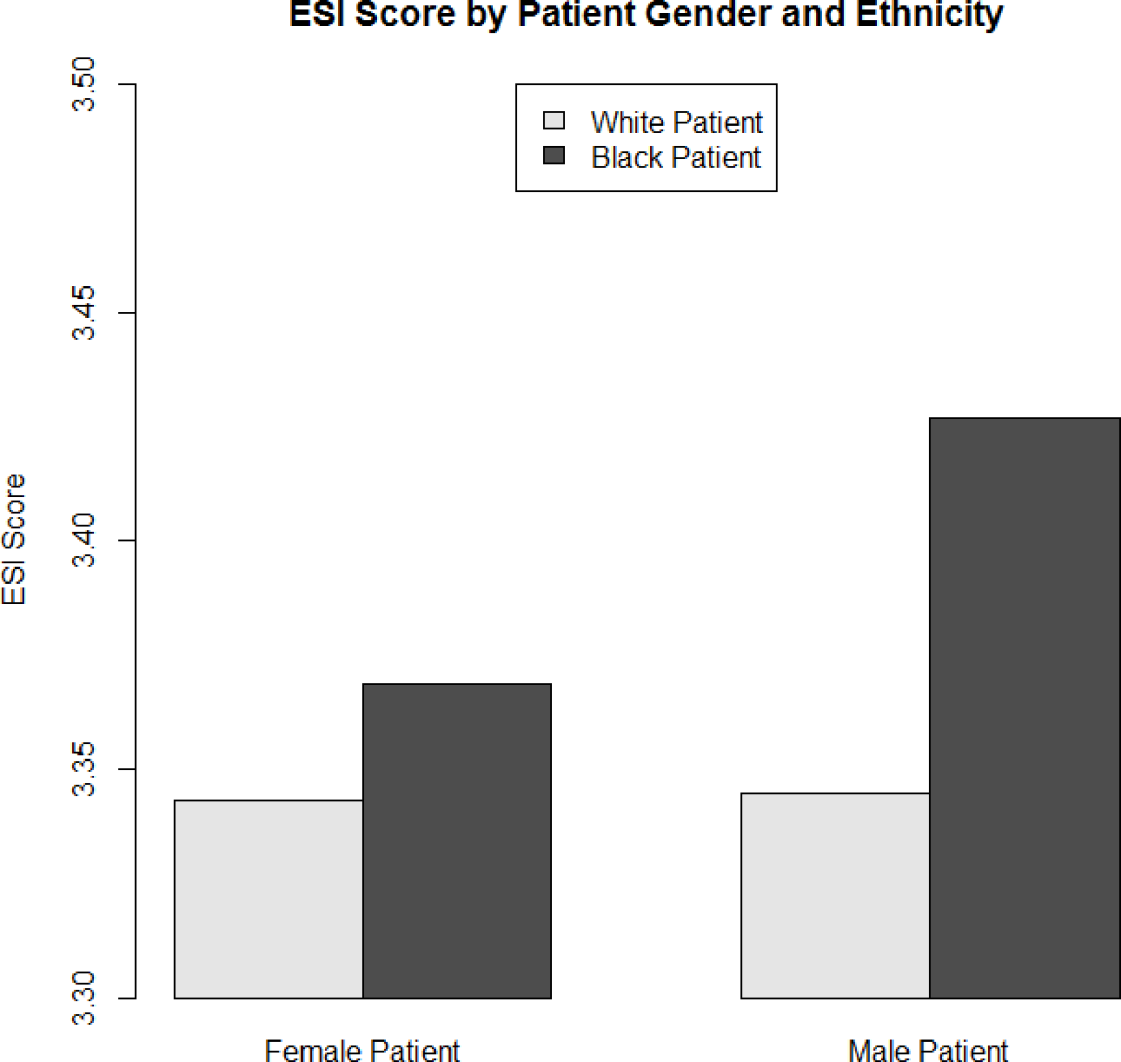
Predicted ESI score by patient ethnicity (x axis) and gender (bars) for full sample when all other covariates are at their mean and problem behaviors is set to zero.

Characteristics of nurses accounted for a greater proportion of variability in ESI (21%) than demographic characteristics and vital signs of patients (13%). In the full sample, there was an interaction between patient gender and the dummy code representing Black patients (Table 2). A simple-slope analysis revealed that Black patients were ascribed less urgent ESI scores than White patients after controlling for patient and nurse characteristics, and the difference between White and Black patients was larger among male patients (*B* =. 08 [*SE* =. 004], *z* = 23.55, *p* <. 001) than among female patients (*B* =. 03 [*SE* =. 011], *z* = 2.36, *p* =. 018; Fig 1). Hispanic patients were given less urgent ESI scores than White patients, but controlling for patient and nurse characteristics, the difference was only significant among male patients (*B* = 0.01 [*SE* =. 006], *z* = 2.04, *p* =. 041), not among female patients (*B* = -.03 [*SE* =. 020], *z* = 1.48, *p* =. 140).

**Table 2:** Results of the linear cross-classified random-effects model predicting ESI from patient and nurse characteristics.

Predictor Level	Predictor	Estimate (Standard Error)	Wald *z*	*p*-value
—	(Intercept)	3.343 (.047)	71.11	<. 001
Patient	Black	0.026 (.011)	2.36	.018
	Black*Gender	0.056 (.011)	5.03	<. 001
	Asian	0.024 (.034)	0.70	.484
	Asian*Gender	0.011 (.036)	0.32	.749
	AIAN	0.092 (.056)	1.63	.103
	AIAN*Gender	-0.080 (.061)	-1.32	.187
	Hispanic	-0.029 (.020)	-1.48	.139
	Hispanic*Gender	0.042 (.021)	2.02	.043
	Mixed	-0.015 (.035)	-0.43	.667
	Mixed*Gender	0.055 (.037)	1.47	.142
	Patient Gender	0.002 (.007)	0.25	.803
	Patient Age	-0.005 (.0001)	-56.21	<. 001
	Heart Rate	-0.005 (.0001)	-65.92	<. 001
	Respiratory Rate	-0.040 (.0006)	-65.97	<. 001
	Behavioral Problems^†^	-0.011 (.001)	-10.37	<. 001
	Pain Score	-0.004 (.0003)	-12.48	<. 001
Nurse	Nurse Gender	0.023 (.027)	0.85	.395
	Nurse Age	0.002 (.001)	1.88	.060
	Nurse Experience	0.001 (.001)	0.99	.322

^†^ Sum of separate substance or alcohol abuse diagnoses.

Among the other patient characteristics, patient age, heart rate, respiratory rate, problem behaviors, and pain score were all negatively related to the patient’s ESI score; patients with higher levels of these characteristics during visits were given more urgent ESI scores. Among the nurses’ characteristics, and despite the substantial proportion of variance in ESI scores attributable to their characteristics, neither nurses’ gender, age, or years of experience significantly predicted ESI scores in the full sample (see S1 Supplemental Materials for a more detailed breakdown of findings).

## Discussion

The Institute of Medicine, federal agencies, and professional societies have all made reducing treatment disparities a major policy goal [15–20]. Previous studies have not been able to reliably quantify the extent to which subgroups of patients that differ in ethnicity/race may receive disparate ESI assignments due to limitations in sample sizes, statistical techniques, and limitations of the databases used that did not account for repeated measures of individuals. Our study using over 350,000 patient-examiner encounters, and controlling for patients’ vital signs and numerous other characteristics of patients and their medical examiners, showed that ED triage nurses assign significantly different ESI scores to some ethnic minority patient subgroups.

Bivariate analyses (Table 1) showed that all the ethnic minority groups, except for American Indians/Alaskan Natives, had a greater odds of receiving less urgent and prioritized ESI scores than White patients, and these effects were largest for Blacks and Asians. Although bivariate analyses do not control for covariates or the nested structure of the data, previous studies [9,10,21] have used that approach. Therefore, these analyses should facilitate comparisons against those earlier studies. Using techniques that captured the influence of unique characteristics of patients and their examiners, Black VA patients were found to receive significantly less urgent ESI scores than White VA patients, an effect that was stronger for Black males than for Black females. Hispanic males were also more likely to receive less urgent ESI scores than White males.

As expected, patients’ vital signs (heart rate, respiratory rate, and pain score) independently predicted more urgent ESI scores, as did patients’ age and problem behaviors (alcohol/substance-related disorders). By comparison, the influence of Black ethnicity on ESI scores, though small in absolute terms (about 1/10^th^ of a point in an ESI score), emerged as a statistically independent and significant predictor of ESI scoring assignments, in addition to patient’s vital signs (i.e. objective factors that are explicitly intended to influence ESI scores). This study cannot determine whether the observed effects contribute to measurement error, as there was no practical way to qualify the validity of ESI assignments in a study this size. Conventional protocols for establishing optimal treatment decisions such as relying on the subjective judgments of so-called experts (e.g., “the gold standard”) are also subject to individual factors (e.g., age, gender, experience, cultural background) that likely affect how experts stylistically report their judgments [22]. However, because patient ethnicity is an omnipresent contextual factor during all health-examiner/patient encounters, there is justification to warrant further investigations of how patient-level and examiner-level characteristics may influence or interact to influence measurement error when assessing patient conditions in emergency settings. Systematic error in triage assessments may also have downstream consequences for patient treatment satisfaction and trust in the health equity of emergency care.

The second major finding of the current study was the substantial proportion of variance in ESI scores that were attributable to individual differences among the triage nurses, irrespective of patients’ characteristics and presenting conditions. Over 20% of the variance in ESI scores was due to characteristics of the triage nurses, which appears to contradict previous (albeit smaller) studies showing minimal influence of personal characteristics of nurses [9,23,24] and high inter-rater reliability between triage nurses and other types of medical professionals (e.g., prehospital triage, physicians [25,26]. In the current study, the nurses’ gender, age, and years of experience did not independently predict ESI scores. Therefore, it is uncertain which triage examiner characteristics or other social contextual factors (e.g., presence of other people in the room at the time of patient presentation [27]) are most important in influencing ESI assignments.

One significant limitation of our study therefore was the unavailability of additional clinical characteristics of patients, information about contextual factors and personal attributes of the examiners, including the nurses’ ethnicity/race. It is likely that controlling for patient-presented vital signs does not provide an adequate sense of the overall clinical “severity” of any given patient presenting to the ED. Another feature that may limit the generalizability of the findings is that the sample included VA Medical Center patients who are predominantly male and older than the general population, and the lower power associated with the substantially smaller sample of female patients may have affected some of the results. Although the VA health care system provides a rich national database of ED records using relatively uniform and standardized procedures, many VA staff are veterans themselves and may have additional characteristics not representative of community EDs. Finally, there may be alternative techniques for analyzing the current data, including multilevel logistic regressions which could account for the ordinal nature of ESI scores (however attempts to analyze the current data using this technique resulted in estimation difficulties).

We do not know whether the differences in ESI scores given to Black males and females and Hispanic males in this study caused those patients to receive less timely or lower quality care. However, triage scores have been shown to be reliable predictors of hospital admission and death [2], and some studies have shown that Blacks and Hispanics with chest pain receive lower triage priority ratings than Whites, and receive fewer procedures including orders for ECGs, cardiac enzymes [10], cardiac catherization [28,29], and analgesic prescribing [30]. At the very least, we suspect that higher ESI scores on average did result in longer wait times in the ED, which can result in poorer outcomes in a variety of diseases, such as myocardial infarction [31], stroke [32], and sepsis [33].

Our study controlled for illness severity by using vital signs (e.g., heart rate and respiratory rate) measured in triage. Additional research is needed to unpack all the factors that contribute to health disparities in treatment delivery including regional differences in population density [34] and group differences in patient-presenting condition or decision-making. For example, it is possible that Black patients may visit the ED for less urgent medical problems due to cultural differences in health promotion behaviors or perceptions of difficulties in obtaining primary care appointments (but see [35–37]). We are, however, confident that the current findings are not attributable to differences in patient financial status, access to care, or communication difficulties, because the VA health care system affords equal access to eligible patients, and all veterans were sufficiently proficient in English to have been in the armed forces of the United States.

In light of these factors, it is reasonable to assume that normative and reflexive (involuntary) social psychological reaction styles operate alongside reflective, induction-based judgments [38] to influence voluntary treatment-decisions in U.S. EDs. We caution that given the retrospective nature of our data, evidence of systematic differences in ESI scores related to race or ethnicity does not confirm systematic error, nor does it warrant any inference about racist motives or attitudes of health examiners or institutionalized racism (for a systematic review, see [39]). A competing hypothesis would be that systematic error in patient treatment, should it be confirmed by future studies, might be attributable to pervasive tendencies among humans to be implicitly attracted to and express compassion for people with similar characteristics (a phenomenon called homophilic peer preferences [22,40].

### Ethics Statement

An ethics statement was not required for this work.

## Supporting Information

S1 Supplemental Materials (DOCX)
