# Supplementary material for: Ethnic Disparities in Emergency Severity Index Scores among U.S. Veteran’s Affairs Emergency Department Patients
Source: PLoS One. 2015 May 29;10(5):e0126792. doi: 10.1371/journal.pone.0126792 (PMC4449190; doi:10.1371/journal.pone.0126792)
Supplement: S1 Supplemental Materials — (DOCX) [file pone.0126792.s001.docx]

**S1 Supplementary Materials**

Table of Contents

1. Methods……………………………………………………………………………………..….…....pg. 2
   1. Database Management Procedures……………….………….……………………….....…pg. 2
   2. Analyses………………………………………….…….……………………………..……pg. 2
2. Results………………………………………………….…..…..………………………………..…..pg. 2
   1. Results for different U.S. Regions…………….………..……………………………….…pg. 2
   2. Results for Different Patient Diagnoses…….…………………..……………………….…pg. 3
3. Discussion…………..……………………………………………………..…………………….…...pg. 3
4. References………………………………………………………………………………………..….pg. 4
5. Tables………………………………………………………………………………………………..pg. 6
6. eTable1 Results Across Regions………………….…………….…………………………………...pg. 6
7. eTable2 Results Across Diagnoses…………………………….………………….......................….pg. 8

**Investigators:**

Jacob M. Vigil

Joe Alcock

Patrick Coulombe

Laurie McPherson

Mark Parshall

Allison Murata

Heather Brislen

**Methods**

**Database Management Procedures**

The data were extracted for a study of pain assessment that will be reported separately. Twenty-two (17%) out of a total of 130 ED stations throughout the U.S. had identifiable ESI data, and were therefore used in the current study. Visit identifier from the ESI score was used to filter for visits having one or more inflammatory or musculoskeletal diagnoses (ICD9s of 680-698.99 and 710-729.99) in order to limit extraneous sources of variability in pain assessment. Records for unique patients seen at multiple facilities were linked using the scrambled SSN. ESI scores were stored as “Health Factor” observations, and were identified via inspection of “health factor type” names, which varied considerably across facilities, and by using key words (e.g., emergency, severity, index, ESI, level, score, ED, ER, triage, acuity). Patients with more than one ESI score during the same visit (for example, within 10 min., because the patient was assessed by two different nurses) were assigned the latest assessment. Only 5-point scales were included. Further, only patients with an ESI score between 2 and 5 were included in the current analyses; ESI Level 1 (resuscitation) scores were omitted.

Because standardized race categories in the database have evolved over time, outdated categories were mapped into standardized values according to VA guidelines. In addition, Asian, Hawaiian and Pacific Islander data were grouped to minimize losses due to these schema changes over time. Hispanic and non-Hispanic ethnicity declarations are also collected, and so for example a patient may simultaneously self-identify as “Black” and “White” (multi-racial) or as “Asian” and “Hispanic” (race and ethnicity). To calculate ethnic/racial categories, the number of entries for each race and ethnicity declaration made by a patient across all the patient visits (during the 5-year period window) were totaled, and patients’ racial and ethnic identity were determined by the proportion of times patients reported themselves as belonging to any of the five racial (American Indian or Alaskan Native [AIAN]; Asian/Hawaiian or Pacific Islander; Black or African American; White; or declined/unknown) or two ethnic categories (Hispanic or Latino; Not Hispanic or Latino). Patients who reported exclusively White race were subsequently split into Hispanic or non-Hispanic White ethnic categories depending on their self-reported ethnic identity. Patients who reported a single ethnicity/race at least 70% of the time were assigned that ethnic/race category; patients who reported multiple ethnicities with less than a 70% frequency were assigned to a mixed-race category; patients with unknown or declined to answer ethnic and racial identities were excluded from analyses.

Vital signs (heart rate, respiratory rate, and pain score) were chosen using the smallest absolute time difference from the timestamp of the ESI score, as long as they were recorded by the same staff member within 1 hour before or after the ESI score. (Approximately 57% of vital signs used for our analysis were recorded from 0 to 10 minutes after the ESI and 88% within 0 to 30 minutes after.) Patient problem lists were examined for substance-abuse related diagnoses. Patients were flagged as to the total number of distinct ICD9 codes in the areas of alcohol, amphetamines, cannabis, cocaine, opioids, and “other” substances of abuse cumulatively over their history and for problems active and not in remission at the time of the ESI observation.

Patient and staff member age were calculated from date of birth, and gender is listed in source tables; years of staff experience was calculated from the date of employment with the US VA Health Care System. Patients and staff with discrepant gender listings or other information were excluded. In total, 89% of the ESI scores were provided by an examiner with a nursing degree. Visits for which an ESI score was assigned by a person other than a nurse (e.g., nursing assistant, physician, student, resident, therapist), and those in which the examiner could not be reliably determined, were excluded from the analyses. Finally, patients with any missing data or outlying data, including a heart rate below 30 or above 200 or a respiratory rate above 50, were removed from the dataset prior to analysis.

**Analyses**

Methodologically, we treated ESI as a continuous variable instead of ordinal, which prevented us from reporting odds ratios after adjusting for other predictors and could have influenced the significance test of some predictors. We initially attempted different optimizers to run multilevel logistic models, as well as different nesting structures. These models did not converge, compelling a more simplified analysis that treats ESI as a continuous variable. It is also important to note that our current focus on patients with an inflammatory or musculoskeletal diagnosis (broad pain-related conditions) might have limited extraneous variability in ESI scores, which may have affected our analyses.

**Results**

**Results for Different U.S. Regions**

We computed the proportion of variance in ESI scores that is due to differences across patients, and the proportion of variance that is due to differences across nurses for different U.S. Census divisions for which there was representative data. The proportions of variance were calculated from a null model with only a random intercept allowed to vary across both patients and nurses. The 22 stations that comprised the usable data were clustered into the regions: Pacific (Colville, WA; Los Angeles, CA; Mather (Sacramento), CA; San Diego, CA; Seattle, WA); Mountain (Albuquerque, New Mexico; Phoenix, AZ; Tucson, AZ); West North Central (Kansas City, MO); East North Central (Cleveland, OH; Dayton, OH; Fort Wayne, IN; Hines, IL); East South Central (Birmingham, Al; Jackson, MS; Montgomery, AL); Middle Atlantic (Buffalo, NY; Pittsburgh, PA; Wilkes-Barr, PA); and South Atlantic (Charleston, SC; Columbia, SC; Huntington, WV). Table S1 (top) shows the proportion of variance attributable to patients and to nurses for each region to examine ethnic differences in ESI scores while accounting for the other covariates.

**Results for Different Patient Diagnoses**

The entire database of useable patient/examiner interactions were further subdivided into patients presenting four broad categories of pain-related musculoskeletal and inflammatory disorders at the time of the triage assessment during the 5-year period examined. The categories were: skin infection (680-689.99), skin inflammation (690-698.99), arthropathies (710-719.99), dorsopathies (720-724.99), and rheumatisms (725-729.99). Table S2 (top) shows the proportion of variance attributable to patients and to nurses for patients with each diagnosis.

**Discussion**

Triage scores, such as the ESI, are meant to prioritize treatments in the setting of resource constraint (e.g. empty ED beds, emergency personnel time, etc.). These assessments also incorporate an estimate of the resource intensity that a patient might require. Although physicians can ultimately determine the degree of resource utilization by a patient, regardless of initial triage score, this score has several concrete effects that predict future treatment: 1) Triage scores can determine whether a patient is referred to an alternate clinical care location, such as urgent care, clinic, or fast track, where available resources are different and patients receive fewer tests over a shorter duration of time, on average; 2) ESI scores influence whether a patient leaves without being seen; 3) Standing orders or nurse initiated protocols may be ordered depending on triage nurse assessment and ESI score; 4) ESI scores make a large impact on the wait times, which are a primary determinant of patient satisfaction with her/his care; and 5) Physicians and midlevel providers will be influenced by the initial triage score to guide decisions about the urgency and intensity of treatment. Systematic differences in ESI scores based on ethnicity and gender are likely to impact all of these areas.^41-45^ Alternatively, while vital signs are objective, anticipating resource needs may be very subjective. Vital signs may actually play a minimal role in ESI assignments, in which case their utility in ED settings may be more limited than is conventionally assumed.

References

1. Tanabe P, Gimbel R, Yarnold PR, Adams JG. The emergency severity index (version 3) 5-level triage system scores predict ED resource Consumption. J Emerg Nurs 2004a; 30: 22-29.
2. Tanabe P, Gimbel R, Yarnold PR, Kyriacou DN, Adams JG. Reliability and validity of scores on the Emergency Severity Index version 3. Acad Emerg Med 2004b;11:59-65.
3. Epps CD, Ware LJ, Packard A. Ethnic wait time differences in analgesic administration in the emergency department. [Pain Manag Nurs](http://www.ncbi.nlm.nih.gov/pubmed/18313587) 2008;9:26-32.
4. Heines JK, Heins A, Grammas M, Costello M, Huang K, Mishra S. Disparities in anlgesia and opioid prescribing practices for patients with musculoskeletal pain in the emergency department. J Emerg Nurs 2006;32:219-24.
5. Okuneri C, Okunseri E, Chilmaza CA, Harunani S, Xiang Q, Szabo A. Racial/ethnic variations in emergency department wait times for nontraumatic dental condition visits in the United States. J Am Dent Assoc 2013;144:828–836
6. Saha S, Freeman M, Toure J, Tippens KM, Weeks C, Ibrahim S. Racial and ethnic disparities in the VA Health Care System: A systematic review. J Gen Intern Med 2008;23:654-671.
7. Todd KH, Deaton C, D’Adamo AP, Goe L. Ethnicity and analgesic practice. Ann Emerg Med 2000;35:11-6.
8. Todd KH, Samaroo N, Hoffman JR. Ethnicity as a risk factor for inadequate emergency department analgesia. JAMA 1993;269:1537-9.

Arslanian-Engoren C. Do emergency nurses' triage decisions predict differences in admission or discharge diagnoses for acute coronary syndromes? J Cardiovascular Nurs 2004;19:280-286.

1. López L, Wilper AP, Cervantes MC, Betancourt JR, Green AR. Racial and sex differences in emergency department triage assessment and test ordering for chest pain, 1997–2006. Academ Emerg Med 2010;17:801–808.
2. Hox JJ. Multilevel analysis: Techniques and applications. New York:Taylor & Francis, 2010.
3. R Core Team R: A language and environment for statistical computing (Version 3.1.0), 2014. (Accessed October 5, 2014, at http://www.r-project.org.)
4. Bates D, Maechler M, Bolker B, Walker S. Linear mixed-effects models using Eigen and S4 (Version 1.1-6), 2014. (Accessed October 5, 2014, at http://CRAN.R-project.org/package=lme4.)
5. Preacher KJ, Curran PJ, Bauer DJ. Computational tools for probing interaction effects in multiple linear regression, multilevel modeling, and latent curve analysis. J Educ Behav Stat 2006;31:437-448.
6. Agency for Healthcare Research and Quality, Rockville, MD. National Healthcare Quality Report 2012, June 2013. (Accessed October 5, 2014, at <http://www.ahrq.gov/research/findings/nhqrdr/nhqr12/index.html>.)
7. Association of American College of Physicians. Racial and ethnic disparities in health care, Updated 2010. Philadelphia: American College of Physicians 2010. (Accessed October 5, 2014, at http://www.acponline.org/advocacy/current_policy_papers/assets/racial_disparities.pdf).
8. American Medical Association. Commission to End Health Care Disparities. Chicago: American Medical Association, 2013. (Accessed October 5, 2014, at http://www.ama-assn.org/resources/doc/public-health/cehcd-strategic-plan.pdf.)
9. National Research Council. How Far Have We Come in Reducing Health Disparities?; Progress Since 2000: Workshop Summary*.* Washington, DC: The National Academies Press, 2012 (Accessed October 5, 2014, at http://iom.edu/Reports/2012/How-Far-Have-We-Come-in-Reducing-Health-Disparities.aspx.)
10. Smedley BD, Stith AY Nelson AR (editors). Unequal treatment: Confronting racial and ethnic disparities in health care. Institute of Medicine (IOM), Committee on Understanding and Eliminating Racial and Ethnic Disparities in Health Care Washington, DC: National Academy Press, 2003. (Accessed October 5, 2014, at http://www.iom.edu/Reports/2002/Unequal-Treatment-Confronting-Racial-and-Ethnic-Disparities-in-Health-Care.aspx.)
11. Ulmer C, Bruno M and Burke S (editors). Future Directions for the National Healthcare Quality and Disparities Reports. Institute of Medicine (IOM), 2010 Washington, DC: National Academy Press, 2010. (Accessed October 5, 2014, at <http://www.iom.edu/Reports/2010/Future-Directions-for-the-National-Healthcare-Quality-and-Disparities-Reports.aspx>.)
12. Saha S, Freeman M, Toure J, Tippens KM, Weeks C, Ibrahim S. Racial and Ethnic Disparities in the VA Health Care System: A Systematic Review. J Gen Intern Med 23(5):654–71
13. Göransson KE, Ehrenberg KE, Marklund B, Ehnfors M. Emergency department triage: Is there a link between nurses personal characteristics and accuracy in triage decisions. Accident Emerg Nurs 2006;14:83-88.

Martin A, Davidson CL, Panik A, Buckenmyer C, Delpais P, Ortiz M. An examination of ESI triage scoring accuracy in relationship to ED nursing attitudes and experience. J Emerg Nurs 2013;40:461-468.

[Buschhorn HM](http://www.ncbi.nlm.nih.gov/pubmed?term=Buschhorn%20HM%5BAuthor%5D&cauthor=true&cauthor_uid=22244546), [Strout TD](http://www.ncbi.nlm.nih.gov/pubmed?term=Strout%20TD%5BAuthor%5D&cauthor=true&cauthor_uid=22244546), [Sholl JM](http://www.ncbi.nlm.nih.gov/pubmed?term=Sholl%20JM%5BAuthor%5D&cauthor=true&cauthor_uid=22244546), [Baumann MR](http://www.ncbi.nlm.nih.gov/pubmed?term=Baumann%20MR%5BAuthor%5D&cauthor=true&cauthor_uid=22244546). Emergency medical services triage using the emergency severity index: is it reliable and valid? [J Emerg Nurs](http://www.ncbi.nlm.nih.gov/pubmed/22244546) 2013;39:e55-63.

1. Esmailian M, Zamani M, Azadi F, Ghasemi, F. Inter-rater agreement of emergency nurses and physicians in Emergency Severity Index (ESI) triage. Emergency 2014; 2:158-161.
2. Vigil, JM, Coulombe P. Biological sex and audience affects pain intensity and observational coding of other people's pain behaviors. Pain 2011;152: 2125–2130.
3. Chen J, Rathore SS, Radford MJ, Wang Y, Krumholz HM. Racial differences in the use of cardiac catheterization after acute myocardial infarction. *New Eng J Med* 2001;344:1443-1449.
4. Schulman KA, Berlin JA, Harless W, Kerner JF, Sistrunk S, Gersh BJ, et al. The effect of race and sex on physicians' recommendations for cardiac catheterization. New Eng J Med 1999;340:618–626.
5. Tamayo-Sarver JH, Hinze SW, Cydulka RK, Baker DW. Racial and ethnic disparities in emergency department analgesic prescription. Am J Public Health 2003;93:2067-2073.
6. De Luca G, Suryapranata H, Ottervanger JP, Antman EM. Time delay to treatment and mortality in primary angioplasty for acute myocardial infarction every minute of delay counts. Circulation 2004;109:1223-1225.
7. Lees KR, Bluhmki E, von Kummer R, Brott TG, Toni D, Grotta JC, et al Time to treatment with intravenous alteplase and outcome in stroke: an updated pooled analysis of ECASS, ATLANTIS, NINDS, and EPITHET trials. Lancet 2010;375:1695-1703.
8. Gaieski DF, Mikkelsen ME, Band RA, Pines JM, Massone R, Furia FF, et al. Impact of time to antibiotics on survival in patients with severe sepsis or septic shock in whom early goal-directed therapy was initiated in the emergency department. Crit Care Med 2010;38:1045-1053.
9. Sonnenfeld N, Pitts SR, Schappert SM, Decker SL. Emergency department volume and racial and ethnic differences in waiting times in the United States. Med Care 2012;50: 335–341.

Lopez JP, Burant CJ, Siminoff LA, Kwoh CK, Ibrahim SA. Patient perceptions of access to care and referrals to specialists: a comparison of African-American and white older patients with knee and hip osteoarthritis. J Natl Med Assoc 2005; 97:667-673.

Washington DL, Harada ND, Villa VM, Damron-Rodriquez J, Dhanani S, Shon H, Makinodan T. Racial variations in Department of Veterans Affairs ambulatory care use and unmet health care needs. [Mil Med](http://www.ncbi.nlm.nih.gov/pubmed/11901574) 200;167:235-41.

Washington DL, Villa V, Brown A, Damron-Rodriguez J, Harada N. Racial/ethnic variations in veterans' ambulatory care use. [Am J Public Health](http://www.ncbi.nlm.nih.gov/pubmed/16257951) 2005;95:2231-7.

Cahan A, Gilon D, Manor O, Paltiel O. Probabilistic reasoning and clinical decision-making: Do doctors overestimate diagnostic probabilities? QJM. 2003; 96: 763-769.

1. [Paradies Y](http://www.ncbi.nlm.nih.gov/pubmed?term=Paradies%20Y%5BAuthor%5D&cauthor=true&cauthor_uid=24002624), [Truong M](http://www.ncbi.nlm.nih.gov/pubmed?term=Truong%20M%5BAuthor%5D&cauthor=true&cauthor_uid=24002624), [Priest N](http://www.ncbi.nlm.nih.gov/pubmed?term=Priest%20N%5BAuthor%5D&cauthor=true&cauthor_uid=24002624). A systematic review of the extent and measurement of healthcare provider racism. [J Gen Intern Med](http://www.ncbi.nlm.nih.gov/pubmed/24002624) 2014;29:364-87.
2. Vigil JM. A socio-relational framework of sex differences in the expression of emotion. Behav Brain Sci. 2009; 32: 375-390
3. Vigil JM, Venner KL. Prejudicial behavior is more closely linked to homophilic peer preferences than it is to trait bigotry. Behav Brain Sci 2012, 35, 38-39.

41. Daudelin DH, Selker HP. Medical error prevention in ED triage for ACS: Use of cardiac care decision support and quality improvement feedback. Cardiol Clin 2005;23:601-614.

# 42. Derlet RW, Kinser D, Ray L, Hamilton B, McKenzie J. Prospective identification and triage of nonemergency patients out of an emergency department: A 5-year study. Ann Emerg Med1995;25:215-223.

# 43. Lucas J, Batt RJ, Soremekun OA. Setting wait times to achieve targeted left-without-being-seen rates. Amer J Emer Med 2014;32:342-345.

44. Pope JH, Aufderheide TP, Ruthazer R, Woolard RH, Feldman JA, Beshansky JR, et al. Missed diagnoses of acute cardiac ischemia in the emergency department. New Eng J Med 2014;342:1163.

45. Retezar R, Bessman E, Ding R, Zeger SL, McCarthy ML. The effect of triage diagnostic standing orders on emergency department treatment time. Ann Emerg Med 2011;57:89-99.

**eTable 1**

**Sample Sizes, Proportions of Variance, and Results of the Cross-Classified Random-Effects Model for Each Region**

|  |  | **E. North Central** | **E. South Central** | **Middle Atlantic** | **Mountain** | **Pacific** | **S. Atlantic** | **W. North Central** |
| --- | --- | --- | --- | --- | --- | --- | --- | --- |
| **Sample Sizes** | **Visits** | 82,905 | 72,647 | 4,760 | 45,970 | 115,402 | 18,519 | 19,439 |
|  | **Patients** | 27,507 | 20,758 | 3,926 | 16,729 | 42,318 | 9,812 | 9,232 |
|  | **Nurse** | 135 | 136 | 84 | 134 | 221 | 83 | 25 |
| % of var. in ESI due to differences across patients | | 14% | 6% | 11% | 13% | 9% | 15% | 26% |
| % of var. in ESI due to differences across nurses | | 18% | 31% | 29% | 11% | 14% | 22% | 19% |
| **Predictor Level** | **Predictor** |  |  |  |  |  |  |  |
| — | (Intercept) | 3.431*** | 3.414*** | 3.285*** | 3.173*** | 3.395*** | 3.370*** | 3.784*** |
| Patient | Black | 0.033 | 0.006 | -0.095 | 0.085* | 0.026 | 0.084* | -0.025 |
|  | Black*Gender | 0.039 | 0.060** | 0.232* | -0.033 | 0.071** | 0.047 | 0.090* |
|  | Asian | -0.058 | 0.110 | -0.038 | -0.086 | 0.047 | 0.118 | 0.149 |
|  | Asian*Gender | 0.013 | -0.078 | 0.059 | 0.156 | -0.004 | -0.056 | -0.145 |
|  | AIAN | -0.298 | -0.458 | 0.048 | 0.261** | 0.082 | 0.097 | 0.163 |
|  | AIAN*Gender | 0.247 | 0.606* | N/A† | -0.236* | -0.070 | -0.201 | -0.156 |
|  | Hispanic | -0.009 | -0.133 | -0.056 | 0.010 | -0.042 | -0.149 | 0.044 |
|  | Hispanic*Gender | 0.002 | 0.203 | -0.022 | 0.029 | 0.036 | 0.255 | 0.057 |
|  | Mixed | -0.124 | 0.032 | 0.383 | 0.149 | -0.069 | 0.044 | -0.081 |
|  | Mixed*Gender | 0.173 | 0.015 | -0.278 | -0.263* | 0.128* | 0.071 | 0.161 |
|  | Patient Gender | 0.016 | -0.038* | -0.096* | 0.035* | 0.010 | 0.043 | -0.060** |
|  | Patient Age | -0.007*** | -0.004*** | -0.001 | -0.005*** | -0.006*** | -0.006*** | -0.004*** |
|  | Heart Rate | -0.005*** | -0.003*** | -0.002*** | -0.004*** | -0.007*** | -0.006*** | -0.003*** |
|  | Respiratory Rate | -0.046*** | -0.022*** | -0.034*** | -0.028*** | -0.050*** | -0.064*** | -0.087*** |
|  | Problem Behaviors | -0.026*** | 0.003 | -0.006 | -0.032*** | -0.003 | -0.008* | -0.007 |
|  | Pain Score | 0.004*** | -0.015*** | -0.022*** | 0.004*** | -0.005*** | 0.007*** | -0.013*** |
| Nurse | Nurse Gender | -0.091 | -0.200 | 0.018 | 0.079* | -0.057 | 0.216 | 0.109 |
| eTable 1 cont. |  |  |  |  |  |  |  |  |
|  | Nurse Age | -0.004 | 0.008*** | 0.004 | 0.015*** | -0.005** | -0.040*** | 0.006 |
|  | Nurse Experience | 0.007* | 0.013** | 0.011* | -0.013*** | 0.011** | 0.049*** | 0.006 |

*Note*. Proportions of variance due to patients and nurses were calculated from a null model with only a random intercept allowed to vary across both patients and nurses.

† This interaction term could not be computed because there were no female AIAN patients in this region.

**p* < .05. ***p* < .01. ****p* < .001.

**eTable 2**

**Sample Sizes, Proportions of Variance, and Results of the Cross-Classified Random-Effects Model for Each Diagnosis**

|  |  | **Skin Infection** | **Skin Inflammation** | **Arthropathies** | **Dorsopathies** | **Rheumatisms** |
| --- | --- | --- | --- | --- | --- | --- |
| **Sample Sizes** | **Visits** | 7,836 | 2,009 | 15,352 | 16,854 | 1,461 |
|  | **Patients** | 6,253 | 1,829 | 11,582 | 11,779 | 1,377 |
|  | **Nurse** | 312 | 202 | 386 | 373 | 185 |
| % of var. in ESI due to differences across patients | | 14% | 12% | 8% | 10% | 3% |
| % of var. in ESI due to differences across nurses | | 17% | 24% | 31% | 27% | 32% |
| **Predictor Level** | **Predictor** |  |  |  |  |  |
| — | (Intercept) | 3.478*** | 3.681*** | 3.525*** | 3.468*** | 3.618*** |
| Patient | Black | 0.167** | -0.063 | 0.013 | -0.011 | -0.028 |
|  | Black*Gender | -0.066 | 0.139 | 0.036 | 0.044 | 0.067 |
|  | Asian | 0.068 | 0.008 | -0.009 | 0.135 | 0.573 |
|  | Asian*Gender | -0.131 | -0.007 | 0.098 | -0.044 | -0.431 |
|  | AIAN | 0.374 | 0.065 | -0.178 | -0.186 | 0.285 |
|  | AIAN*Gender | -0.508* | 0.166 | 0.167 | 0.144 | -0.296 |
|  | Hispanic | 0.175 | -0.178 | -0.122 | 0.060 | 0.269 |
|  | Hispanic*Gender | -0.133 | 0.266 | 0.143 | -0.064 | -0.152 |
|  | Mixed | -0.123 | -0.284 | 0.156 | -0.051 | -0.116 |
|  | Mixed*Gender | 0.191 | 0.125 | -0.108 | 0.056 | 0.132 |
|  | Patient Gender | -0.011 | 0.033 | 0.035 | 0.078*** | 0.049 |
|  | Patient Age | -0.005*** | -0.004*** | -0.003*** | -0.004*** | -0.004*** |
|  | Heart Rate | -0.005*** | -0.004*** | -0.002*** | -0.002*** | -0.002* |
|  | Respiratory Rate | -0.029*** | -0.031*** | -0.023*** | -0.029*** | -0.048*** |
|  | Problem Behaviors | 0.007 | 0.014 | 0.008** | 0.014*** | -0.012 |
|  | Pain Score | -0.030*** | -0.041*** | -0.007*** | -0.006*** | -0.015** |
| Nurse | Nurse Gender | -0.049 | 0.009 | 0.029 | 0.023 | -0.017 |
| eTable 2 cont. | |  |  |  |  |  |
|  | Nurse Age | 0.006** | 0.008* | 0.007** | 0.007*** | 0.006 |
|  | Nurse Experience | 0.002 | 0.002 | 0.004 | 0.005 | 0.004 |

*Note*. Proportions of variance due to patients and nurses were calculated from a null model with only a random intercept allowed to vary across both patients and nurses.

**p* < .05. ***p* < .01. ****p* < .001.
